# Psychological distress and associated factors among South Asian individuals with chronic obstructive pulmonary disease: a convergent mixed methods study

**DOI:** 10.1515/med-2026-1384

**Published:** 2026-02-25

**Authors:** Hussan Zeb, Ahtisham Younas, Angela Durante, Ercole Vellone

**Affiliations:** Nursing, Swat College of Nursing, Swat, Pakistan; Faculty of Nursing, Memorial University of Newfoundland, St. John’s, Canada; Interdisciplinary, School of Advanced Studies Sant’Anna, Pisa, Italy; Department of Biomedicine and Prevention, University of Rome Tor Vergata, Rome, Italy; Nursing Department, Biruni University, Istanbul, Turkey; Department of Nursing Science, Faculty of Health Sciences, University of Eastern Finland, Kuopio, Finland; Division of Research Methodology, Department of Nursing, Faculty of Nursing and Midwifery, Wroclaw Medical University, 51-618 Wrocław, Poland

**Keywords:** COPD, psychological distress, sociocultural, South Asians, anxiety, depression

## Abstract

**Objectives:**

To develop a comprehensive understanding of psychological distress and associated sociocultural and disease related factors among South Asian Individuals with COPD.

**Methods:**

A convergent mixed methods study was used. The quantitative sample included 408 and qualitative sample included 22 individuals (11 patients and 11 family caregivers). Hospital Anxiety and Depression Scale was used for quantitative data collection and semi-structured interviews for qualitative phase. Descriptive and regression analyses were used for quantitative data and reflexive thematic analysis for qualitative data. Qualitative and quantitative data were integrated using merging integration procedure and statistics-theme joint display.

**Results:**

The mean depression score was 10.91 ± 2.42 and the anxiety score was 9.91 ± 2.69. Quantitative and qualitative integrated data showed that factors affecting psychological distress included sex, education level, primary decision maker in the household, years of living with COPD, financial constraints, fears of the future, self-care struggles, interrupted family decision-making process, and relational support.

**Conclusions:**

Individuals with COPD reported moderate to high levels of psychological distress influenced by societal, family, and disease related factors. Community based programs are also needed for providing adequate mental health and counselling resources to patients and their family caregivers.

Chronic Obstructive Pulmonary Disease (COPD) is characterized by a progressive irreversible limitation in airflow. The condition primarily stems from exposure to risk factors such as cigarette smoke, environmental pollutants, and occupational hazards [1]. COPD is a leading global health concern, and it is projected that by 2030, it may become the third leading cause of death worldwide [2]. Al Wachami (2024), based on a meta-analysis of 42 studies, noted a global prevalence of COPD was 12.64 % (N=339,475) [3]. Pakistani population is disproportionately affected by COPD due to extensive exposure to biomass fuel, air pollution, and tobacco usage [4]. Approximately 90 % of COPD-related deaths occur in low- and middle-income countries (LMICs) including Pakistan highlighting the socioeconomic and healthcare disparities that exacerbate the condition’s impact [5], 6]. In Pakistan, COPD is still underdiagnosed and inadequately treated, which significantly lowers patients’ quality of life and raises related healthcare costs [7]. This warrants the need for studying South Asian Pakistani popualtion with COPD. Various systematic and integrative reviews highlighted that COPD can negatively impact physical, emotional, and mental health of individuals with disease and their formal and informal caregivers, leading to psychological distress [7], [8], [9]. Psychological distress is a general negative mental state characterized by an individual’s perceived inability to manage stress. It often manifests as mild-to-moderate symptoms of depression and anxiety, which, if left unrecognized, may progress to more serious mental health conditions [10]. Previous research on psychological distress is mainly quantitative and focuses on multiple chronic diseases [8], [11], [12], [13], [14], [15]. There is a dearth of qualitative studies for comprehensive understanding of psychological distress and its associated sociocultural and disease related factors in diverse contexts. Therefore, this mixed methods study was designed to gain both qualitative and quantitative understanding of psychological distress and its associated factors among patients with COPD.

## Background

Individuals with COPD experience persistent disease and symptom burden [9], 10]. These individuals are more likely to seek consistent healthcare services and avoid social networking because of their disease, resulting in social isolation and mental health problems [16], 17]. COPD impacts individuals’ physical health by reducing their lung function, and also contributes to psychological distress due to its chronic and progressive symptom burden and impact on life [18]. Empirical research demonsrated that individuals with COPD are more likely than the general population to have psychological discomfort or other mental health issues [19], [20], [21]. Persistent psychological distress can worsen clinical symptoms, reduced sleep quality, diminished self-management skills, and decrease quality of life of those with COPD [5], 20], 21].

Previous studies demonstrated that psychological distress is linked to chronic disease [17], [22], [23], [24] and the level of distress is associated with the type of chronic illness, housing circumstances, educational attainment, years of living with a chronic illness, relationships within the family, and available support systems all had a direct effect on psychological distress [12], 14], 15]. Additionaly, sociocultural and disease related factors can also contribute to psychological distress in chronic diseases [12], 15]. Therefore, there is a need to better understand the various sociocultural, disease and societal factors affecting psychological distress in COPD across diverse contexts and populations. Previous quantitative and qualitative research explored sociocultural and disease related factors of psychological distress in individuals with psoriasis [2], chronic kidney disease [25], [26], [27], diabetes [21], 28] and a combination of cardivascular, cancer, and respiratory chronic diseases [12], [13], [14], [15, 29]. Research on socioctural factors and psychological distress among individuals with COPD is mainly quantitative in nature and included individuals from Europe, United States, China, UK, Grecce, Korea, Japan, and Canada [8]. There is a dearth of qualitative studies for comprehensive understanding of psychological distress and its associated sociocultural and disease related factors in other diverse contexts such as South Asia and other low and middle income countries. Gaining this understanding is critical to ensure that the increasing burden of COPD, distress, and its impact can be addressed through exploring contexualized and unique factors for specific populations. Therefore, in the absence of qualitative studies on psychological distress and its associated factors in global and particulary South Asian context, this mixed methods study was designed to fill this gap.

## Purpose

To develop a comprehensive understanding of psychological distress and associated sociocultural and disease related factors among South Asian Individuals with COPD. The specific questions were:

1. What are the levels of depression and anxiety among individuals with COPD? (Quantitative)
2. What sociocultural factors affect anxiety and depression among individuals with COPD (e.g., age, sex, family system, type of health care services)? (Quantitative)
3. What societal and disease related factors do individuals with COPD and their family caregivers perceive as influencing anxiety and depression? (Qualitative)
4. To what extent do the quantitative data about levels and factors affecting anxiety and depression converge with or diverge from the qualitative data about individuals and family caregivers perceived factors affecting their psychological distress? (Mixed Methods)

## Methodology

A convergent mixed methods design (QUAL + QUAN) involving parallel data collection and analysis was used [30], 31]. Both qualitative and quantitative phases were given equal weight and leveraged the strengths of both methods to develop a comprehensive understanding of psychological distress and associated factors.

### Setting and context

The data were collected at five large tertiary care hospitals in two cities (Peshawar and Swat) from June 2022 to January 2023, in Khyber Pakhtunkhwa, Pakistan. These hospitals had 14 outpatient departments, and about 500 individuals with COPD visited these departments every week.

### Sample and sampling

The quantitative sample size was calculated using the formula (n=z^2^ [p × q] / d^2^), where n=sample size, p=estimated proportion of the main study variable was approximate (50 %), q=1 – p (50 %), and d=margin of error (5 %). The estimated sample size was 408. A convenience sampling technique was used because of the lack of sampling frame as the exact number of patients and their list could not be obtained from the outpatient units.

Patients were selected based inclusion criteria: a) at least 30 years of age at any stage of COPD, b) patients with confirmed diagnosis of COPD, c) patients care for self at their homes or communities, and d) patients who showed an interest to participate and were able to provide informed consent. The patients’ diagnosis of COPD was confirmed after reviewing their medical records at the clinics. Medical records indicated that spirometry was used at the time of admission as a diagnostic test to assess airflow obstruction as per Global Initiative for Chronic Obstructive Disease (GOLD)’s diagnostic criteria of forced expiratory volume in 1 s (FEV1)/forced vital capacity (FVC) less than 0.7 after bronchodilator use [32]. For qualitative phase, both patients and family caregivers were invited for interviews. The inclusion criteria for family caregivers was: a) caring for family members with COPD in home or community settings, b) accompanying their relatives to clinic or hospital visits, c) able to provide written informed consent.

### Data collection instrument

Demographic information was collected using a questionnaire consisting of 13 items about age, gender, ethnicity, language, socioeconomic status, educational status, type of family, primary decision maker in the family, type of community, type of chronic illness, number of years living with chronic illness, and type of health care services utilized. Psychological distress was measured using the Hospital Anxiety and Depression Scale (HADS). The HADS was developed by Zingmond and Snaith and comprises two subscales and 14 items [33]. The Hospital Anxiety and Depression Scale Depression Subscale [HADS-D]) and the Hospital Anxiety and Depression Scale Anxiety Subscale [HADS-A]).

A three-point Likert scale is used as the response set: ‘0’ (not at all) to ‘3’ (most of the time). Items 1, 3, 5, 7, 9, 11, and 13 are designed to assess HADS-A and items 2, 4, 6, 8, 10, 12, and 14 to determine HADS-D. The total HADS-A and HADS-D score is the sum of all the items. For scoring, eight items are reversed scored (1, 3, 5, 11, 13, 6, 8, 10). The total score for the sub scales ranges from 0 to 21 (0–7=normal, 8–10=borderline, and 11–21=either anxious or depressed) [33]. The Urdu-translated HADS developed by Lodhi et al. (2020) was used in this study [34]. The Urdu version has acceptable validity and reliability. In this study sample, the Cronbach’s alpha for the anxiety subscale was 0.82 and 0.64 for the depression subscale.

### Data collection process

Data were collected from June 2022 to January 2023. Posters and outreach to patient support groups were key recruitment strategies. The researcher (HZ) dropped off the printed scale and his contact information at the outpatient clinics, and the nurses in the clinics invited patients to complete the printed scale. They also asked patients to contact the researcher if they would like to participate in in-depth interviews. The researcher visited the hospitals biweekly to collect the completed scale. Before data collection, the participants received complete written and verbal study information from the nurses, and they were encouraged to ask questions to the researcher using the provided contact information. The nurses who helped in data collection received training in data collection. The participants were asked to provide their written consent after being fully informed about the study.

Those patients and family caregivers who indicated interest in participating in interviews, were contacted to set up time and place for the interviews. Before interviews, the researcher (HZ) provided detailed study information and its purpose to each participant and caregiver in plain native language (Pashto or Urdu). The interviews were conducted separately as well as together for both patient and caregivers as per their choice to ensure that participants are comfortable during interviews. All interviews were audio-recorded and transcribed verbatim in Urdu and/or Pashto and English. The average length of the interviews was 41 min.

When using Reflexive Thematic Analysis (RTA) for analysis, Braun and Clarke [35], advise against aligning with arbitrary rules of saturation, but encourage to provide an explicit definition of saturation and pragmatic decisions surrounding sample size. Our sample size was based on availability of patients with COPD and their caregivers. As Braun and Clarke [35], recommended, we provide an explicit definition of data saturation as assessed in this study. We defined data saturation as the degree to which research participants shared similar ideas or views during interviews as expressed by other participants in previous interviews [36]. After nine interviews, patients and their caregivers seem to share similar information. We conducted two additional interviews (4 in total for patients and caregivers) to ensure that saturation was in fact achieved. Hence, data collection was stopped at 22 interviews. During parallel data collection and analysis of qualitative data, similar codes were apparent which further validated that data saturation has been achieved.

### Data analysis

The data were analyzed using Social Package for the Social Sciences (SPSS) 26.0. Descriptive statistics were calculated for the items such as means and standard deviations. Multiple linear regression analyses were completed to determine the factors associated with psychological distress. The choice and entry of variables (including age, sex, socioeconomic status, family type, living community, education level, type of health care services used, years of living with COPD, and number of family caregivers) in the regression analysis was based on previous studies among South Asians with COPD and chronic conditions [15], 37], 38] .Variance Inflation Factor (VIF) and tolerance are used to detect multicollinearity and P-value less than 0.05 was considered as significant.

As previously stated, RTA was used for qualitative analysis [39], because it allows for exploring patterns of shared meanings of individuals’ experiences [40]. RTA is a theoretically flexible method entailing several paradigmatic orientations such as inductive, deductive, semantic, latent, and critical, and constructionist [35]. We used inductive orientation to stay close to the data and generate a rich narrative of the participants’ experiences [40]. The data were analyzed using MAXQDA Analytics Pro.

We followed the following steps for RTA a) a careful and thorough reading of verbatim transcripts several times to develop an understanding of the overall meaning of the participant’s views, b) data coding using semantic (i.e., coding exact words and phrases of participants) and latent (i.e., going beyond the apparent meaning of words and phrases) codes were developed which is consistent with the inductive orientation, c) the generated codes were collated for further refinement based on similar meanings and linkages and thematic maps were created, d) the refined codes were then named and combined into subthemes, and d) the relevant and similar subthemes were combined into final themes. Finally, we interpreted participants experiences by reflecting on the semantic and latent codes and subthemes, and formulated themes that adequately capture the nuanced meaning of their experiences [40]. Mixed methods analysis was completed using statistics-theme joint display was used [41], and the meta-inferences were generated using the seven-step process outlined in Younas et al. [42].

### Rigor

Several measures were used to establish the rigour of individual qualitative and quantitative phases and the overall mixed methods design. For quantitative phase, valid and reliable data collection instrument was used, data collectors were trained, *apriori* sample size estimation was completed, and regression analysis was undertaken based on prior empirical research. For the qualitative phase, to ensure trustworthiness, the researchers accounted for personal biases throughout data collection, analysis, and interpretations, engaged in intrapersonal dialogues and wrote reflective journals, peer debriefing was completed during analysis, and audit trail was maintained by using MAXQDA for analysis [43], 44]. Additionally, premature closure of data was prevented by using first approach which entails in-depth familiarization with the data and segmented analysis that involves analyzing data in segments [45]. Thick description of study was providing by offering a contextual, relational, and emic account of participants views and experiences [46].

To ensure rigour for mixed-methods analysis, data integration was made explicit using the merging integration technique and developing joint displays [30], 31]. In addition, the legitimation criteria [47], 48], was used to address threats to validity of mixed methods. Commensurability approximation legitimation, weakness minimization legitimation, the integration legitimation, multiple validities, and divergent findings legitimation were established. The commensurability approximation pertains to the idea that qualitative and quantitative approaches are commensurable, and effective integration can produce wholistic and more comprehensive meta-inferences, that the inferences drawn from the individual strands [47], 48]. The weakness minimization legitimation pertains to the extent to which the weakness of the qualitative approach is addressed by the strengths of the quantitative approaches and vice versa [47]. In this study, the results of the quantitative phase were complemented by the results of the qualitative phase. The integration legitimation pertains to the extent that the researcher integrates qualitative and quantitative data, this must be supported by metainferences which gives added value beyond the qualitative and quantitative insights [48]. The divergent findings legitimation pertains to the extent which researchers adequately report discordant results after integrated analysis [49].

### Ethical considerations

Ethical Approval was obtained from the Saidu Medical College and Saidu Group of Teaching Hospitals approved the study protocol (IRB#15-ERB/2022). All the patients and family caregivers completed written informed consent. They were assured of their right of privacy, confidentiality, and anonymity. All transcripts and data were coded and kept under locked and only researchers had access to the raw data. The data collection instrument was used after authors’ permission.

## Quantitative findings

### Demographic information

Of 408 enrolled individuals, 72.1 % were male (n=294), and 27.9 % were female (n=114), with a mean age of 56.52 ± 11.13. Most of the participants lived in rural communities (n=228, 59.9 %) and the remaining lived in urban communities (44.1 %, n=180). Most patients identified as Pashtoon (n=379, 92.2 %). Most of the patients identified their socioeconomic status as lower middle class (Rs. 1,166–2,253 [USD 4.17-8.0] income/month/person; n=186, 45.6 %), upper middle class (Rs. 3,808–7,769 [USD 13.62-27.78] income/month; n=107, 26.2 %), and middle class (Rs. 2,253–3,808 [USD 8.0-13.62] income/month; n=73, 17.9 %) (Table 1).

**Table 1:** Demographics quantitative phase.

Variables	Mean (SD)
**Patient age**	56.52 (11.13)
	**Frequencies (%)**
**Patient sex**	
Male	294 (72.1 %)
Female	114 (27.9 %)
**Type of residential community**	
Rural	228 (55.9 %)
Urban	180 (44.1 %)
**Ethnicity**	
Punjabi	10 (2.4 %)
Pashtoon	379 (92.9 %)
Prefer not to say	19 (4.5 %)
**Socioeconomic economic status**	
Upper class (Rs. 7,769/USD 27.70 or higher income/month/person)	21 (5.1 %)
Upper middle class (Rs. 3,808–7,769/ USD 13.62-27.70 income/month/person)	107 (26.2 %)
Middle class (Rs. 2,253–3,808/ USD 8.0-13.62 income/month/person)	73 (17.9 %)
Lower middle class (Rs.1,166-2,253/USD 4.17-8.0 income/month/person)	186 (45.6 %)
Lower class (Rs. 1,166/USD 4.17 or lower income/month/person)	20 (4.9 %)
**Education level**	
No education	126 (30.9 %)
Primary school (Grade 5)	160 (39.2 %)
Middle school (Grade 8)	12 (2.9 %)
High school (Grade 10)	21 (5.1 %)
Bachelor’s degree	65 (15.9 %)
Master’s degree	24 (5.9 %)
**Family type**	
Nuclear	104 (25.5 %)
Joint	304 (74.5 %)
**Primary decision maker in the household**	
Men	378 (92.6 %)
Women	30 (7.4 %)
**Years of living with COPD**	
0–2 Years	184 (45.1 %)
3–4 Years	105 (25.7 %)
5 or above	116 (27 %)
**Smoking habit**	
Smokers	241 (58.8 %)
Non-smokers	166 (41.2 %)
**Number of family caregivers**	
0–2	107 (26.2 %)
3–4	198 (48.5 %)
5 or above	103 (25.2 %)

### Levels of psychological distress

The mean HADS-D score was 10.91 ± 2.42 and the HADS-A score was 9.91 ± 2.69 indicating moderate levels of depression and anxiety. The item with the highest mean score in HADS-A scale was reported for “I can sit at ease and feel relaxed” (1.85 ± 0.92) and “I get a sort of frightened feeling like ‘butterflies’ in the stomach” (1.78 ± 0.96). In HADS-D, the highest mean score was reported for “I still enjoy the things I used to enjoy” (2.15 ± 0.78) and “I can enjoy a good book or radio or TV program” (1.97 ± 0.92) (Table 2).

**Table 2:** Anxiety and depression.

Responses	N	Mean	Std. D
**Anxiety**			
I feel tense or ‘wound up’	408	1.13	0.42
I get a sort of frightened feeling like ’butterflies’ in the stomach	408	1.78	0.96
I get a sort of frightened feeling as if something awful is about to happen	408	1.13	1.02
I feel restless as I have to be on the move	408	1.50	0.84
Worrying thoughts go through my mind	408	1.26	0.89
I get sudden feelings of panic	408	1.26	0.83
I can sit at ease and feel relaxed	408	1.85	0.92
**Depression**			
I feel as if I am slowed down	408	0.68	0.86
I still enjoy the things I used to enjoy	408	2.15	0.78
I have lost interest in my appearance	408	1.43	0.97
I can laugh and see the funny side of things	408	1.77	0.93
I feel cheerful	408	1.39	0.78
I look forward with enjoyment to things	408	1.51	0.83
I can enjoy a good book or radio or TV program	408	1.97	0.92

### Sociocultural factors affecting psychological distress

Regression analysis showed that sex, type of residence, number of family caregivers, and years of living with COPD were positive predictors of depression. The negative predictors of depression included age, ethnicity, type of health care services used, and primary decision maker in the household. The model explained 52 % of the variance [F (18, 356)=23.642, p=<0.001, Adjusted R^2^=0.521] (Table 3). The positive predictors of anxiety included age, sex, type of residence, type of health care, number of family caregivers, and years of living with COPD. The negative predictors of anxiety included education level and primary decision maker in the household. The model was statistically significant and explained 72 % of the variance [F (18, 356) = 53.021, p<0.001, Adjusted R^2^=0.715] (Table 4).

**Table 3:** Regression analysis for depression.

Variables	B	β	p-Value
Age	−0.024	−0.108	0.016
**Sex**			
Female	Reference	Reference	
Male	1.455	0.272	<0.001
**Ethnicity**			
Punjabi	Reference	Reference	
Pashtoon	−3.937	−0.265	<0.001
**Type of residence**			
Urban	Reference	Reference	
Rural	0.558	0.115	0.014
**Socioeconomic Status**			
Upper class	Reference	Reference	
Upper middle class	−0.034	−0.006	0.945
Middle class	0.159	0.026	0.771
Lower middle class	−1.045	−0.217	0.074
Lower class	−1.117	−0.112	0.082
**Education Level**			
No education	Reference	Reference	
Primary (Grade 5)	−3.756	−0.765	<0.001
Middle (Grade 8)	−0.407	−0.030	0.532
High school (Grade 10)	−5.194	−0.498	<0.001
Bachelor’s degree	−5.417	−0.800	<0.001
Master’s degree	−5.843	−0.577	<0.001
**Primary decision maker**			
Women	Reference	Reference	
Men	−2.056	−0.233	<0.001
**Number of family caregivers**			
0–2	Reference	Reference	
3–4	1.162	0.242	<0.001
>4	0.224	0.042	0.706
**Years of living with COPD**			
0–2	Reference	Reference	
3–4	1.740	0.325	<0.001
5 or above	0.237	0.043	0.534
**R** ^**2**^ **: 0.544** Adjusted R Square: **0.521**			

B, unstandardized coefficients; β, standardized coefficients.

**Table 4:** Regression analysis for anxiety.

Variables	B	β	p-Value
Age	0.048	0.195	<0.001
**Sex**			
Female	Reference	Reference	
Male	0.435	0.072	0.055
**Ethnicity**			
Punjabi	Reference	Reference	
Pashtoon	−2.989	−0.178	<0.001
**Type of residence**			
Urban	Reference	Reference	
Rural	1.570	0.285	<0.001
**Socioeconomic status**			
Upper class	Reference	Reference	
Upper middle class	−0.251	0.041	0.562
Middle class	−0.954	−0.140	0.045
Lower middle class	−1.154	−0.213	0.023
Lower class	−0.748	−0.058	0.201
**Education level**			
No education	Reference	Reference	
Primary (Grade 5)	−0.415	0.075	0.057
Middle (Grade 8)	−7.044	−0.460	<0.001
High school (Grade 10)	−1.947	−0.166	0.002
Bachelor’s degree	1.970	0.258	<0.001
Master’s degree	3.460	0.314	<0.001
**Primary decision maker**			
Women	Reference	Reference	
Men	−1.751	−0.176	<0.001
**Number of family caregivers**			
0–2	Reference	Reference	
3–4	3.080	0.571	<0.001
>4	2.686	0.444	<0.001
**Years of living with COPD**			
0–2	Reference	Reference	
3–4	1.790	0.297	<0.001
5 or above	2.166	0.350	<0.001
R^2^: **0.728** Adjusted R Square: **0.715**			

B, unstandardized coefficients; β, standardized coefficients.

## Qualitative findings

In total, 11 individuals with COPD and 11 family caregivers participated in the interviews. Eight patients were male and only three females with the aged ranged from 30 to 65 years. All the family caregivers were male because the participants were recruited from a hospital setting where women generally do not take the role of patient attendant (i.e., family members who accompany their relative to hospital or health care setting) in KPK, Pakistan. All the patients and family caregivers mainly lived in rural settings. Only three of the patients had received some education (Grade 4 or Grade 5) while the remaining were not educated. All the family caregivers were educated (Table 5).

**Table 5:** Demographic patients and family caregivers.

Patients with COPD						
S. no.	Age	Sex	Marital status	Education	Residential community	Yrs. with disease
1	55	Male	Married	Nil	Rural	10
2	65	Male	Married	Nil	Rural	10
3	60	Male	Married	Nil	Rural	10
4	58	Male	Married	Grade 5	Semi-urban	02
5	64	Male	Married	Grade 5	Rural	10
6	60	Female	Married	Nil	Rural	10
7	42	Female	Married	Nil	Eural	03
8	53	Male	Married	Nil	Urban	06
9	45	Male	Married	Nil	Rural	04
10	59	Male	Married	Grade 4	Rural	10
11	30	Female	Married	Nil	Rural	02

**Family caregivers**						

1	34	Male	Married	Primary	Rural	
2	40	Male	Married	Grade 8	Rural	
3	20	Male	Single	Intermediate	Rural	
4	28	Male	Married	Grade 10	Semi-urban	
5	40	Male	Married	Primary	Rural	
6	30	Male	Married	Grade 8	Rural	
7	20	Male	Married	Grade 5	Rural	
8	30	Male	Married	Grade 10	Urban	
9	26	Male	Married	Grade 10	Rural	
10	24	Male	Single	Diploma/ Intermediate	Semi-urban	
11	42	Male	Married	Grade 3	Rural	

## Themes

Five themes were generated to capture the factors that exacerbated and/or prevented the development of distress among individuals with COPD and their family caregivers. Each of the factors is described in detail as follows.

### Self-care struggles

Self-care struggles of individuals with COPD was discussed as a factor exacerbating psychological distress. Both individuals with COPD and their family caregivers discussed various types of struggles such as inability to perform activities of daily living, engaging in family and social activities, managing disease, and symptom burden, and complications. Family caregivers shared that their loved ones rely on them, often completely or partially, for performing activities of daily living which lead to feelings of powerlessness, fatigue, distress, and emotional suffering within the family. This in turn, results in creating a stressful environment for the family and loved ones with COPD. For example, one female patient shared how COPD has affected her life, leading to deteriorating self-care and increasing stress and anxiety in her life.

This breathing illness has finished my life. Nowadays I can’t do anything, I am burden on my family. I used to do all sorts of activities; I have done farming, have helped my husband in construction of our house, but now I just do small things, like washing the utensils and nothing else. The struggle is real and difficult, and I cannot even take care of myself, and it troubles me (Female, 42 yrs).

Family caregivers shared that they had witnessed the self-care struggles of their loved ones firsthand. Many discussed that their family members with COPD consider themselves a burden on the family and expressed that they want to escape from this suffering. However, they need to rely on family to manage the disease burden effectively. Family caregivers shared that this has contributed to immense emotional distress in the family. One of the caregivers shared how seeing his brother struggling troubles him and affects the brother and their family.

It’s sometimes very disheartening for me to see my brother struggling for life. I sometime think how our lives are short, and we are all mortal. There was a time that my brother used to be very strong and was able to carry out all his activities without any support from us, he has now become our weakness. But then I think that this is the best opportunity to honor the services of my brother and helping him in his self-care is important for me. I know that it is difficult for him, he does not want help, but he needs it (Brother, caregiver, 34 yrs).

### Financial constraints

Financial constraints were among the most frequently discussed sources of distress among individuals with COPD and their caregivers. The participants shared that most of them had slender means and lived in poverty in rural areas. It is for this reason that they worked as laborers in coal mines. The disease had also drastically affected family resources and finances needed for meeting basic needs of the family and the individual with COPD. Caregivers whose loved ones with COPD were the primary financial source for the family expressed that they continuously battling their poverty because the family member who earned most money had fallen sick. Many family caregivers worked extra hours to provide for their family, but they also noted that due to increasing inflation, unemployment, and increasing caregiving and family demands, they are unable to provide the needed resources. For example, one of the caregivers shared how resources have depleted and how he is working extra hours, but he is still unable to meet the needs of the family.

We are struggling to afford two meals a day. Medicine prices are increasing daily. We are unable to pay the electricity bills. I work very hard to provide for the family. In the government hospitals, nothing is available. We have to purchase all the medicines from private medical stores. This time when we came to the hospital, we had to borrow the money as well. These financial problems are one of the causes of stress in the family (Caregiver, Male, 20 yrs).

All the individuals with COPD had lost their jobs due to deteriorating health, had limited funds available to support themselves and their families. These individuals shared that self-care, treatment, and medications were no longer affordable for them which caused a great deal of stress in their lives. They noted that they did not have adequate funds to buy and renew medications, afford follow-up visits and travel to the hospitals during emergency situations. One of the patients shared how he had lost his job, and his children are also struggling for employment, which results in financial suffering for the family.

I worked as a laborer in the coal mine due to poverty and no education. I have a big family; my children are also struggling for employment. My relatives try to help me, but as you know in this high inflation everyone is suffering. Due to my hospital admission, my sons have been staying with me, and they are unable to do their daily work due to which they are also losing their daily earnings. This hospital is also very far away from my home, which also cost us a lot. We have to spend money on transportation, and we have to eat from hotels which is too much expensive for us. All the medicine and treatment are also very expensive. These financial problems have crippled me and my family and lead to a lot of stress (Male, 55 yrs).

## Fears for the future

Individuals with COPD and their caregivers expressed concerns and fears about their future given the debilitating effects of disease on their lives. Participants shared that while they try to manage the distress associated with disease burden, treatment, and other social and familial impact, but often they feel uncertain about the future. They indicated being worried about themselves, their families, their family lives, their finances, and the potential effect of treatment on their well-being, as well as possible disease complications. Many participants also shared existential thoughts about their future and wished early death to prevent their families from burden. They noted that being a burden on their families is hard and affects them emotionally resulting in more intrusive and existential thoughts. One of the patients shared how disease impacted him and how it has contributed to worries about the future, thereby worsening his stress and mental well-being.

This disease has taken everything from me. I am not sure what my future will be like. I pray that one day I will recover, but I know this might not happen. I worry about my family and how they will live once I am no longer in this world. I suspect that things will get worse for me and my family, but I also want to get better. These kinds of thoughts are worrisome for me (Male, 45 yrs).

Family caregivers also expressed that having a loved one with COPD had affected them mentally, often leading to feelings of hopelessness about the future and powerlessness to control their lives. They shared that they emotionally support each other during tough times, but sometimes negative thoughts, feelings, and emotions are overwhelming and increase their stress about their loved ones and their families. One of the caregivers provided a detailed account of how his father’s illness has affected him and how he worries about his father and family.

The illness of my father has affected me physically and mentally. Like, sometimes I am uncertain and not sure what will happen in the future. Me and my family are worried about our father and ourselves. You know, fear is there all the time, but sometimes it is overwhelming. COPD, the breathing disorder is various serious, and sometimes I fear I will lose my father. When I am on duty and receive a call regarding my father illness it’s too much painful, when I am with my father everything is in front of me so I can manage accordingly. But when I am far away it is very challenging and different thoughts come to my minds. Then, I call my family members and friends and give them instructions on how to care for my father (Son, Caregiver, 24 yrs).

### Interrupted family decision making process

Individuals with COPD and their family caregivers discussed that the family decision making process was interrupted and contributed to stress within the family. They shared that interruption in family decision making was attributed to several reasons including role reversal, inability of family members to undertake their routine household tasks, financial issues, communication issues, and medical problems of other family members. Many individuals with COPD were heads of their families and took the lead in decision making regarding family affairs. However, due to their illness they had limited ability to participate in decision making and were dependent on other members for their well-being. This often led to interruption in the usual functioning of the family and contributed to stress. One of the caregivers shared how his father’s illness and lack of ability to decision making had resulted in family suffering.

My father used to be the head of family. He used to manage many things for our family and was responsible for making decisions as the head of the household. So, losing his health has affected him and he cannot make the decision like he used to, which means the family is suffering a lot (Son, caregiver, 20 yrs)

Individuals shared that they missed being able to contribute to family affairs by offering material resources such as money as well as playing role in making decisions with the family about ongoing and urgent matters. They expressed feelings of sadness, disappointment, and hopelessness about not being able to share the family work. One of the patients shared how illness and dependency on other family members have affected him and his ability to perform his role in the family.

When I was healthy, I was performing all my work like earning, making decisions, fulfilling my household responsibilities. I felt I was an important member of my family. I was able to go anywhere I wanted. I used to perform all my daily activities independently. I used to practice my religious activities. I used to sit with my family members and often discussed many family issues. But now I am confined to bed and home, and it has made me dependent on others. This dependency is not easy to manage, and I feel powerless in controlling my own life and my family (Male, 60 yrs)

### Relational support

Relational support which entailed mutual and reciprocal of family members for each other was seen as a positive factor in preventing stress and distress within the family. Individuals with COPD and their caregivers expressed that familial support was encouraging and motivating for them and helped them build resilience to manage the negative impacts of disease. The family caregivers noted that they often praised each other for good work and encourage their loved ones with the illness to continue performing self-care as needed. This family connectedness and cohesion was seen as a strategy to alleviate their distress. One of the care givers elaborated how they adjust and prioritize their responsibilities in the family and seek help and support when needed, which eventually helps them in combating stress.

One big challenge is managing my father’s daily needs while also doing other responsibilities like work and taking care of other family members. Then I have adjusted my schedule and prioritized tasks. Sometimes seek help from other family members or friends to share the workload. We try to help each other physically and emotionally when we can and when we need each other. This helps in managing the stress related to care, disease management and household responsibilities (Son, caregiver, 24 yrs).

Individuals with COPD expressed that always having family support in homes and hospitals had greatly reduced their stress pertaining to self-care, family management, and disease burden. They noted that extended families, acquaintances, and friends also offer physical, financial, and moral support when needed; hence reducing their stress. Family caregivers and individuals with CODP shared that they rely on each other at all times and share each other’s burden to address the negative effect of the disease on their lives. One of the patients shared how her family came together as a whole to support her and each other during the times of needs.

In the home, my married daughters visit me regularly. They come more often when I am sick, and they take care of me like helping me in bath, eat, clean, and other activities as well. My son is here to help me all the time. We all help each other. I rely on their help completely, but my sons, daughters-in law, and married daughters, they all rely on each other for money, transport, and household work (Male, 45 yrs).

## Integrated results

Integration of quantitative and qualitative data revealed many confirmed (i.e., consistent qualitative and quantitative findings), expanded (i.e., qualitative and quantitative findings provided additional insights), and discordant (i.e., inconsistent qualitative and quantitative findings) metainferences (Creswell and Plano Clark, 2018). As illustrated in the joint display (Table 6), both qualitative and quantitative data showed moderate to high levels of psychological stress among individuals with COPD. Qualitative data from family caregivers confirmed that they witnessed increasing distress among their loved ones with disease. Financial constraints, self-care struggles, and fears of the future were identified as factors increasing the distress among both family caregivers and individuals with COPD. The quantitative regression analysis showed that living with COPD and living in rural settings were significant predictors of anxiety and depression. Qualitative data showed that low socioeconomic status was identified as a factor leading to increased distress, but this was a non-significant predictor in quantitative regression analysis. This could be because we financial struggles are much more than merely annual household income and we only measured socioeconomic status using household income in the quantitative data. However, in the qualitative interviews participants disucssed at length how lack of money affected their childcare, lives, family relations, access to services, and health care management. Therefore, this was a discordant result. Qualitative data generated two additional insights (expanded) about potential factors such as interrupted family decision making process and relational support as increasing and preventing psychological distress.

**Table 6:** Statistics-theme display of psychological distress and associated factors.

Quantitative Findings	Qualitative Findings	Meta-inferences
The mean HADS-D score was 10.91 ± 2.42 and the HADS-A score was 9.91 ± 2.69 indicating moderate levels.	Both individuals with COPD and their family caregivers shared that living with the disease contributed to greater distress within the family. ***Supporting quote*** “This disease has brough a lot of physical and emotional suffering to me and my family. We struggle to keep up with life” (Patient 4).	**Confirmed:** Individuals with COPD experience psychological distress which also contributes to distress among family caregivers.

**Factors Associated with Psychological Distress**		

Living in rural settings was a significant predictor of HADS-A (B=1.570, β=0.285, p= <0.01) and HADS-D (B=0.558, β=0.115, p=0.014). The demographic data also shows that large number of participants lived in rural settings (55.9 %) and belonged to lower middle (45.6 %) or lower socioeconomic class (4.9 %).	**Financial Constraints:** Living with rural settings, low socioeconomic status, and disease induced financial constraints contributed to psychological distress among patients and their family caregivers. ***Supporting quote*** “I was the only source of income for my family. After my illness we have spent all the saved money. I have borrowed money from my family members and friends. But as you know the increasing inflation is crippling everyone in the country inflation. The prices of medicines are almost triple from last one to two years. We cannot manage our expenses” (Patient 8).	**Expanded:** Among two indicators of poverty (socioeconomic status, rural settings), rural settings was a significant predictor. However, qualitative data confirmed both of these as possible predictors of psychological distress. Additionally, having a family member with COPD aggravated the financial situation/poverty of the families leading to distress. **Discordant:** Socioeconomic status was not a significant predictor of psychological distress. It could be because we only measured it using one indicator.
Living with 3-4 years with COPD was a significant predictor of HADS-D (B=1.740, β=0.325, p= <0.01). Living with 3–4 (B=1.790, β=0.297, p= <0.01) and 5 or above years (B=2.166, β=0.350, p= <0.01) with COPD was significant predictor of HADS-A.	**Self-Care Struggles:** Exacerbating COPD contributed to increased self-care struggle for the individuals and the families in supporting self-care. This lead to increased psychological distress among individuals with COPD. **Fears for the Future:** Exacerbating COPD also resulted in increased uncertainties and fears for the future among individuals with COPD and their family caregivers. ***Supporting quote*** “Caring for someone with this disease is not easy. It is very difficult for me and my family. There are times when it feels devastating, like when she has a really hard time breathing, I fear she might not survive. These moments put a lot of pressure on me, my wife and my daughter. I fear what will happen, if my wife is no longer with us; the life will become impossible for me. I fight with these worries and thoughts almost every day” (Husband, Caregiver 11).	**Confirmed:** Individuals with COPD experience increased psychological distress as their disease exacerbated. Increased exacerbations lead to increased self-care struggles and fears and uncertainties about the future, which in turn contributed to stress.

HADS-D, hospital anxiety and depression-depression; HADS-A, hospital anxiety and depression-anxiety; B, unstandardized coefficients; β, standardized coefficients.

## Discussion

To the best of our knowledge, this was the first mixed methods study that investigated the psychological distress among South Asian individuals with COPD. The core findings of the study are that these individuals experienced moderate to high level of psychological distress, and this distress was further exacerbated by several sociocultural, disease and family related factors such as sex, education level, poverty, interrupted family process, years of living with COPD, number of family caregivers, and the relational support available within families. The results direct our attention to significant mental health concerns for individuals with COPD and their family caregivers. These findings are consistent with prior research that emphasizes the psychological burden associated with chronic illnesses such as COPD and cardiovascular disorders in Asian context [15], 50].

In our study the mean HADS-D score was 10.91 ± 2.42 and the anxiety score was 9.91 ± 2.69 which are somewhat consistent with prior studies [14]. For example, two prior studies noted that depression and anxiety often occur simultaneously in patients with COPD, with prevalence estimates of 26–43 % [51], 52]. In our study, significant predictors for depression included gender, education level, primary decision maker in the household, smoking, and years of living with COPD. These results highlight the multifaceted nature of psychological distress in COPD patients and the importance of addressing sociodemographic factors in treatment plans. The significant predictors for anxiety included gender, type of residence, primary decision maker in the household, type of health care community, years of living with COPD, and number of family caregivers. These finding are also consistent with [15] who identified that factors such as living conditions, educational level, family dynamics, and available support systems significantly influence psychological distress and coping strategies. Additionally, Pollard et al. highlighted that factors such as food instability, housing, unemployment, and transportation can hinder access to quality healthcare and exacerbate psychological distress among individuals with chronic conditions [53].

Given these findings, the study underscores the importance of community-based support approaches to address these factors such as living conditions, educational level, family dynamics, and available support systems to mitigate psychological distress and promote healthy coping among patients and their caregivers. Qualitative data showed that family caregivers also experienced distress while caring for their loved ones. However, in South Asian context the critical role of family caregivers is often unrecognized, and they fail to get the needed support and resources to engage in effective caregiving [37], 54]. The study underscores the importance of community-based support approaches to address the self-care needs of individuals with COPD and the caregiving needs of their family caregivers. Health and social care professionals need to consider these broader social factors when treating and caring for patients with COPD. Future research is warranted to collaborative work with health and social care professionals to develop and evaluate culturally sensitive and tailored caregiver support programs, coping and disease management programs, and ani-poverty interventions for individuals with COPD and their family caregivers.

Financial constraints emerged as a major theme, showing how financial difficulties affect day-to-day living in an extensive way. Other research has also found similar results, highlighting how people’s capacity to pay for healthcare, maintain a healthy lifestyle, and meet basic requirements is severely hindered by their poor financial means [55], 56]. Individuals with COPD also reported difficulties in sustaining self-care, which typically led to declining physical and mental health and increased distress. Previous studies [57], 58] indicate that patients with chronic illnesses typically struggle to sustain self-care routines due to a lack of time, finances, distress, and motivation. Self-care is more challenging for those belonging to socioeconomically underprivileged communities (such as South Asians in this study) [59].

Support from relationships was recognized as a coping strategy as well as a challenge. While some participants felt neglected by their social circles, others emphasized the importance of emotional and practical support from family and friends. Prior studies have emphasized the protective role of social support in reducing stress and enhancing health status and well-being [60], 61]. Therefore, it is critical that health and social care professionals work with these individuals and their family caregivers to ensure that care in homes and communities is effective and sustainable. Health care centers in the communities can develop partnerships with health care professionals in acute care settings to design social and mental health support programs for patients and their family caregivers. Further research can focus on co-designing and testing the effectiveness of social support programs for this population. Further research is also warranted to develop more comprehensive understanding of qualitative factors affecting psychological distress mainly interrupted family decision making process and relational support. A greater understanding of these factors can be useful for developing social support, caregiving, and effective coping programs for individuals with COPD and their family caregivers.

### Limitations

There are several limitations of this study. First, our sample was convenience, participants were mainly recruited from Northern Pakistan, caregivers were primary male due to cultural factors, and individuals and caregivers belonged to lower middle to middle income class. Future research should aim to include a more representative and diverse sample to enhance the generalizability of results. Therefore, the results have limited generalizability in other contexts with different populations characteristics. However, results are useful for generating further hypotheses for testing. Second, we defined socioeconomic status mainly based on the monthly household income. It should be noted that socioeconomic status can also be affected by factors such as number of family members in the household, employment status of family members, transportation, distance from health care facilities, and availability of governmental financial aid programs. Furthermore, the reliance on a single, self-reported income variable may have contributed to measurement error and diminished the statistical power to detect the true impact of socioeconomic status in the quantitative models. Future studies should use additional factors for measuring socioeconomic status to achieve a more robust and multi-dimensional representation of the construct. Third, there is also the possibility of self-selection bias and only interested participants came forward for interviews whose perspective may differ from those who declined to participate. These limitations may affect external validity of the study. Finally, the quantitative sample only included individuals with COPD and no family caregivers participated in the survey. Future studies should consider including both patients and their caregivers in both qualitative and quantitative phases.

## Conclusions

Individuals with COPD reported moderate to high levels of psychological distress influenced by a range of societal, family, and disease related factors. Therefore, there is a need for health and social professionals and institutions to collaborate with family caregivers and design community-based social and caregiving support programs. Community based programs are also needed for providing adequate mental health and counselling resources to patients and their family caregivers. Local governments in South Asian context need to develop policies and ani-poverty programs to address the financial constraints of patients and their caregivers. Relational support and interrupted family decision making process are important factors to be considered when offering health and social care support to South Asian immigrants living with COPD and their family caregivers in high income countries.
